# Are we accepting enough? Examining the association between homophobia and acceptance of diversity

**DOI:** 10.1177/27526461241297383

**Published:** 2024-10-30

**Authors:** Farhin Chowdhury, Yasemin Erdogan, Chiaki Konishi

**Affiliations:** Department of Educational and Counselling Psychology, 5620 McGill University, Montreal, QC, Canada

**Keywords:** Homophobia, school bullying, adolescents, acceptance, sexual diversity

## Abstract

Peer victimization is a serious public health problem and Dan Olweus, pioneer in bullying research, has long recognized school safety as a fundamental human right. The purpose of this study was to assess and address a facet of a larger and more complex critique on issues related to Equity, Diversity, and Inclusion in schools. As such, the present study examined how peer and adult acceptance of sexual diversity is associated with homophobic perpetration and peer victimization among high-school students. Two hundred nineteen participants (61.3% boys, *M*_age_ = 14.82, *SD* = 1.31) across high schools in Southern Quebec, Canada, completed questionnaires assessing adult acceptance of sexual orientation and reports of homophobic perpetration and victimization among students. Results from path analyses revealed that adult acceptance of sexual diversity significantly predicted homophobic perpetration, *β* = −0.221, *t* (9) = −2.68, *p* = .002, and victimization, *β* = −0.193, *t* (9) = −3.05, *p* = .007, while peers’ acceptance of sexual diversity did not. The findings highlight the importance of adult acceptance of sexual diversity in reducing peer victimization, emphasizing the need for a discrimination-free school climate.

## Introduction

“Well, I have been criticized and been called a ‘faggot’ so much, I don’t know. Whenever I hear that word ‘faggot’ it strikes fear into my heart. I start getting nervous” (Butler et al., 2003: 11).

This participant’s report is an example representing many youths’ lived experiences of homophobic peer victimization, the frequent actions or lack of actions surrounding these incidents, and the consequences.

Feelings of belonging, safety, and acceptance are important for all students, particularly for those vulnerable to homophobic victimization (Allen et al., 2018; Black et al., 2012). Dan Olweus, pioneer of bullying research, defines bullying as an intentional negative action inflicted repeatedly in an imbalanced power relationship (Olweus, 2013). Recognizing school safety as a fundamental human right, Dan Olweus has long emphasized its importance. The Universal Declaration of Human Rights Act, which ensures the right to education free from discrimination of any kind, along with the Canadian Human Rights Act, which is founded on the principles of equality and freedom from discrimination, guarantees students the right to an education free from discrimination and harassment, regardless of their gender identity or sexual orientation. Despite these protections, sexual minority students (e.g., lesbian, gay, and bisexual; LGBTQ+) as well as those who deviate from heterosexual norms continue to face homophobia in schools across the globe (Moyano and Sánchez-Fuentes, 2020). For the purpose of this study, we refer broadly to sexual minority youth to include individuals with a continuum of self-identified sexual orientation (e.g., mostly heterosexual and bisexual) and distinguish them from heterosexual youth to examine victimization based on their *perceived* sexual orientation.

Indeed, homophobia is deeply ingrained in contemporary society and permeates through the social and physical spaces of schools (Cénat et al., 2015; Taylor and Peter, 2011). Homophobia is defined as prejudice and discrimination against a group of people or an individual on the basis of their perceived sexual orientation and/or gender identity (Major et al., 2002). This includes discrimination against anyone who is, or is perceived as being, LGBTQI (lesbian, gay, bisexual, transgender, queer, and intersex). In schools, as in broader society, heterosexual hegemony is assumed and perpetuates inequality, with most homophobic attitudes being based on the principle of heteronormativity (Bhana, 2012). Unfortunately, individuals who deviate from heterosexual societal standards^1^ are frequent targets of homophobic victimization. Additionally, derogatory terms such as “gay”^2^ and “lezzie” are often used to reinforce traditional gender norms, extending their harmful impact beyond just sexual minority students (Mays and Cochran, 2001). While blatant, overt, or explicit forms of homophobia still exist, subtle forms can also manifest through microaggressions. Homophobic microaggressions are commonplace, occurring with or without the intention of harm in everyday interactions. These can take various forms, such as verbal microaggressions (e.g., saying “that’s so gay” to describe something as “weird”) and the denial of heteronormativity by asserting that schools are “neutral.” These subtle behaviors are deeply ingrained in daily interactions, making it difficult to recognize such harassment and its impact (McCabe et al., 2013). It is important to highlight that homophobic peer victimization can take many forms, including physical, verbal, and relational forms of victimization (Steven et al., 2014). Peer victimization along with the passive acceptance of homophobic behavior from school members contributes to the persistence of homophobic school climates (Steven et al., 2014). For instance, disregarding acts of homophobic violence from school staff and failing to protect a victim may constitute discrimination. Thus, adult school members including teachers, counselors, and administrators can also be complicit in encouraging homophobic behavior (Birkett et al., 2009; Johnson, 2014), fostering a discriminatory and unsupportive environment for many adolescents (Konishi et al., 2013). This homophobic environment, characterized by discrimination and victimization experiences, can put students at physical and psychological risk, as well as negatively impact their academic achievement (Marshal et al., 2011; Steven et al., 2014; Walch et al., 2016). However, social support such as from teachers and peers has been found to mitigate these negative outcomes (Espelage et al., 2008). Therefore, it is imperative to leverage previous studies on social support, particularly acceptance, and examine the factors that may reduce homophobic behavior in schools among adolescents. As such, the present study intended to investigate the association between acceptance of sexual diversity from both adults and peers and homophobic behavior (i.e., victimization and perpetration) among high-school students in Southern Quebec.

### Negative implications of homophobia

Homophobia has numerous adverse consequences on *all* students, particularly on sexual minority students (Moyano and Sánchez-Fuentes, 2020; Peter et al., 2015). For example, Peter et al. (2015) found that homophobic victimization (i.e., physical and verbal abuse) can put both heterosexual and sexual minority adolescents across Canada at risk for negative outcomes, although the effects were far more severe for sexual minority students. Notably, their results showed that verbal and physical victimization can lead to more severe forms of homophobic bullying among both sexual minority and heterosexual youth in high schools (Peter et al., 2015). Hence, students can face homophobic bullying based on their *perceived* or *actual* sexual orientation. Further, extensive research has documented that both victimization and perpetration have adverse consequences on students’ academic achievement, social-emotional well-being, and physical health (AntÓnio and Moleiro, 2015).

In regard to academic outcomes, sexual minority and heterosexual students who felt unsafe at school due to the homophobic culture were more likely to skip classes and dislike school, have lower levels of educational aspirations and school engagement, and be at a greater risk for school dropout (Konishi and Saewyc, 2014; Steven et al., 2014; Taylor and Peter, 2011). Perpetrators of homophobic behavior also exhibited poorer school performance, reporting lower grades and lower self-perceived academic competence (Hysing et al., 2019; Ma et al., 2009).

Concerning health outcomes, victimization has been associated with negative mental outcomes, namely, internalization of homophobia (Walch et al., 2016), depression and anxiety, suicidal ideation, and substance misuse among adolescents (Denny et al., 2016; Wilson and Cariola, 2019). Homophobic perpetration was associated with poor health-related quality of life, depression and anxiety (Albaladejo-Blázquez et al., 2019) as well as externalization symptoms such as aggression among adolescents (AntÓnio and Moleiro, 2015; Kelly et al., 2015; Olweus, 1997). Moreover, homophobia has physical health implications. For instance, victims of homophobia are at a greater risk of experiencing threats of physical and sexual assaults (Meyer, 2010), while perpetrators are at a risk of psychosomatic symptoms, injuries, and other physical health symptoms (Stein et al., 2006; Wolke et al., 2001).

Taken together, adolescents, whether victims or perpetrators, may be at a higher risk of the aforementioned negative outcomes. However, a positive school environment that is accepting and supportive consistently has been documented to protect students from the harmful effects of homophobic behavior in school (Espelage et al., 2008).

### Importance of acceptance of diversity

The importance of an inclusive school climate has garnered significant attention in the bullying literature revealing that a positive school climate is associated with a host of positive outcomes for students, such as academic, social, and behavioral outcomes (Gage et al., 2019). Part of a positive school climate includes the level of acceptance of diversity. As Meyer (2010) has noted, “every school community has elements of diversity in it, however, the level to which it embraces and recognizes this diversity as valuable or negligible is important” (p. 9). Thus, equity, diversity, and inclusion are important principles that must be *encouraged* and *embraced* by school members to ensure that students of diverse sexual-orientation backgrounds have equal opportunities to thrive in school. Indeed, people’s acceptance of diversity contributes to how said spaces are determined and how students may see themselves in relation to their belonging, safety, and possible futures (Allen et al., 2018; Arens and Niepel, 2019). Although many are resilient and show healthy development outcomes, sexual minorities report greater feelings of unsafety and lower sense of belonging at school than heterosexual peers (Pistella et al., 2020; Poteat et al., 2014). In fact, previous research has indicated that poorer perceptions of respectful classroom norms and lower sense of belonging were associated with greater engagement in homophobic behavior among adolescents (Poteat et al., 2013; Poteat and Russell, 2013). However, an inclusive school environment rooted in acceptance of diversity, generally, has been associated with less bullying involvement, more school connectedness, greater perceived school support, as well as reduced suicide attempts among sexual minorities (Carney et al., 2018; Green et al., 2021; Holt and Keyes, 2003; Proulx et al., 2019). What is more, an inclusive school climate rooted in acceptance of diversity has positive implications for *all* students, extending beyond sexual minority youth (Birkett et al., 2009; Proulx et al., 2019; Rinehart et al., 2016). For instance, the study by Birkett and colleagues (2009) found that a positive school climate that does not tolerate homophobic harassment was associated with lower levels of substance use, depression, and anxiety for both sexual minority and heterosexual students. Although the literature has documented the association between social support (i.e., from peers and adults) for the overall health and well-being of youth, there is mixed evidence on whether the general support can attenuate the effects of homophobic victimization (Poteat et al., 2014). Instead, it has been suggested that the impact of such victimization can be better attenuated with sexual-orientation specific support (Poteat et al., 2014). Research suggests that sexual-orientation specific support can be beneficial in creating a safer environment for sexual minority youth to seek help from adults and peers for concerns related to their sexual orientation and experiences of victimization related to homophobia (Doty et al., 2010; Poteat et al., 2014). As such, more research has delved into examining the importance of *sexual* diversity climate specifically.

A sexual diversity climate ensures the safety of all students, including sexual minority youth, and encourages awareness and support from all school members (Szalacha, 2003). Such sexual diversity climate can be defined as “the quality of the internal environment of the school experienced by students regarding the level of safety, tolerance, and atmosphere of respect for sexual minority individuals” (Szalacha, 2003: 62). Fortunately, research shows that attributes of school climates are not set in stone and can change through efforts. For example, the implementation of an inclusive school policy can lead to positive differences in the school climate, especially policies that specifically protect students from peer victimization and harassment (Szalacha, 2003). It must be noted that, although systemic changes are effective, the changes at the individual level are where it all starts. That is, members of the school community must play an active role in creating an environment that is welcoming and accepting of all students. The educators and school community must understand and address issues of gender and sexual diversity in schools (Meyer, 2010). Considering the literature on acceptance of diversity, it is therefore imperative to take a closer look at homophobia in the context of contemporary schools and better understand the factors promoting a safer school climate. Specifically, one that encourages acceptance of gender and sexual diversity and promotes the well-being and safety of all students. Some studies have provided evidence for the protective role of social support from peers and adults in the face of peer victimization (Olweus and Limber, 2010; Schacter et al., 2021).

#### Role of peer and adult support

Acceptance and social support are closely interconnected, as outlined by Sarason’s (1990) theoretical model, which highlights that “acceptance” is an integral aspect of social support. Sarason emphasizes that acceptance encompasses feelings of love, value, and being accepted by significant individuals in one’s life. In the school setting, peers, teachers, and adult staff constitute the microsystem in which youth are situated in and can influence their sense of acceptance. Thus, in the context of the school environment, both peers and adults play a key role in adolescents’ development (Olweus, 1995). The school community such as peers and adults play a key role on youth’s development and outcomes—a sense of acceptance being a shaping factor in this process. Naturally, the main source of social support for individuals would come from those who frequently surround them; for school-aged children, that includes their fellow peers.

During school years, peers and peer interactions become particularly salient (Rubin et al., 2008). Through this, the support and acceptance that is sourced from peers can be very powerful, especially among peer victimized youth (Schacter et al., 2021). Research on peer acceptance finds that it is linked to higher self-esteem, academic achievement, and prosocial behavior (Bishop and Inderbitzen, 1995; Wentzel and Caldwell, 1997). The existing literature on acceptance and support suggests that high levels of peer support and trust are associated with lower homophobic bullying (D’Urso and Pace, 2019) and serve as a protective factor against externalizing and internalizing problems (Fite et al., 2019; Hodges et al., 1999). For instance, findings from a study by AntÓnio and Moleiro (2015) revealed that sexual minority students in high school who were victims of homophobic bullying reported lower emotional distress when social support (i.e., family and peer support) was higher. However, it is noteworthy to mention that considerable variations in findings across studies still exist and it is important to consider *when* and *for whom* peer support benefits victimized youth (e.g., Schacter et al., 2021). Nevertheless, support from peers can be a critical resource as it can play a protective function in the face of homophobic victimization (Schacter et al., 2021).

As with the frequent presence of peers in schools, another potentially salient source of social support for youth is adults in schools. As youth spend a significant amount of time at school and are surrounded by teachers and other school personnel (i.e., teachers and administrative staff), their experiences with these adults can shape various outcomes. In fact, caring interactions with adults in school can lead to children’s improved sense of belonging and value (Kirby and Gibbs, 2006). While the research on adult social support is not as extensive as that of peer social support, it is nevertheless evident that caring support from adults in school is effective in reducing some concerning outcomes, such as engaging in risky behavior, while helping with positive outcomes, such as liking school (Konishi and Saewyc, 2014). Indeed, the teachers’ and other school personnel’s acceptance are also important factors in students’ development. The existing but limited literature suggests that adult acceptance in schools is associated with students’ school attitudes, school belongingness, and overall well-being (Allen et al., 2018; Arens and Niepel, 2019; Ibrahim and El Zaatari, 2020). Thus far, research on general support and acceptance, particularly from peers, has been presented. However, much less is known on the relations between adult acceptance of *sexual* diversity and homophobia in schools. Thus, although extensive literature has examined peer acceptance of diversity (e.g., D’Urso and Pace, 2019), that of adult school members (e.g., teachers and administrators) is lacking. Indeed, adults such as teachers at school play an important role in the structuring of the social environments in schools that impact adolescents’ outcomes (Olweus, 1995).

### The current study

This study is unique in its specific focus on the role of acceptance of *sexual* diversity in homophobic bullying. It contributes to the limited body of research examining the role of adult acceptance of diversity in schools for youths of all sexual orientations, whilst adding to the extant literature on the roles of peer acceptance. As such, the goal of this study was to examine whether acceptance of sexual diversity can help reduce homophobia among students of diverse sexual orientations. Specifically, the study aimed to examine whether peer and adult acceptance of sexual diversity can help reduce homophobia among students of diverse sexual orientations. The following research question was examined: are peer acceptance of sexual diversity and adult acceptance of sexual diversity, both, independently associated with homophobic perpetration and homophobic victimization among high-school students in Quebec? We hypothesized that both peer and adult acceptance of sexual diversity significantly predicted homophobic perpetration and victimization among students based on the previous studies (AntÓnio and Moleiro, 2015; Moyano and Sánchez-Fuentes, 2020; Olweus, 1995), controlling for factors such as grade and sex. These covariates were selected because of the documented grade and sex differences in correlates of homophobia (e.g., Birkett and Espelage, 2015).

## Methods

### Participants

Participants included 234 participants from high schools throughout southern Quebec in Canada. Specifically, participants were English-speaking adolescents in Grades 8 to 11 between the ages of 13 and 19 (*M*_age_ = 14.76, *SD* = 1.32). The sample (61.3% boys) consisted of students who identified on a continuum of sexual orientations (e.g., completely or mostly heterosexual, completely or mostly homosexual, and bisexual) with 80% identifying as completely heterosexual (see Table 1). Prior to data collection, parental consent and adolescent written assent were obtained. Participants completed a questionnaire assessing (1) demographic information, (2) perceived adult- and peer-acceptance of sexual diversity, (3) reports of homophobic perpetration, and (4) homophobic victimization.

**Table 1.** Descriptive statistics of sexual orientation identifications among participants.

Sexual orientation	Frequency	Percent (%)
Completely heterosexual	188	79
Mostly heterosexual	20	8.5
Bisexual	13	5.5
Mostly homosexual	3	1.3
Completely homosexual	4	1.7
Not sure	7	3

### Procedure

Prior to the administration of the survey, the study was reviewed and approved by McGill University’s Research Ethics Board Office and the school district board. Participants were recruited from one high school with the assistance of the school guidance counselor, who facilitated the distribution and collection of consent and assent forms. Parental consent was obtained for all student participants included in this study. After explaining the purpose and nature of the study to students in eighth- through eleventh-grade classrooms, interested students were asked to take home to their parent letters detailing the research purpose, requirements for consent, and the confidentiality of students’ responses. Students were given the same information as part of the written instructions for the survey and the assent form in person. After obtaining both parental consent and student assent, participants were asked to complete the survey anonymously within a single-class period lasting 30–50 min and were assured of the confidentiality of their individual responses. After completing the survey, students were invited to seek professional support if they experienced any distress or needed someone to talk to. An optional page was included at the end of the survey, allowing students to request help. They could fill in their name and grade, indicating their desire for support. This information would be passed to the school counselor, who would then contact the students. The page was destroyed after the information was passed on, ensuring confidentiality. This process ensured that students had access to support if the survey triggered any distress.

### Measures

#### Demographic characteristics

To obtain descriptive information about the sample, participants were asked to provide information on their age, grade, biological sex (i.e., male or female), and sexual orientation (e.g., completely heterosexual, bisexual, and mostly homosexual; Saewyc et al., 2004).

#### Adult acceptance of sexual diversity

Students’ perceptions of adult (i.e., teachers, administrators, and other school personnel) acceptance of sexual diversity were assessed using the following single item: “adults at my school are accepting of all individuals regardless of their sexual orientation” on a seven-point Likert scale, ranging from 1 = *disagree strongly* to 7 = *agree strongly* (Konishi et al., 2017). A higher score indicated higher levels of adult acceptance of sexual diversity. Among the current sample, Cronbach’s alpha (α) was 0.94, indicating high reliability.

#### Peer acceptance of sexual diversity

Students’ perceptions of peer acceptance of sexual diversity were also assessed using the following single item: “students at my school are accepting of all individuals regardless of their sexual orientation” on a seven-point Likert scale, ranging from 1 = *disagree strongly* to 7 = *agree strongly* (Konishi et al., 2017). A higher score indicated greater peer acceptance of sexual diversity. Cronbach’s alpha (α) was 0.90, suggesting high reliability.

#### Homophobic perpetration

Homophobic perpetration was assessed using seven items asking students about their engagement in homophobic behaviors (e.g., How often have you made someone feel bad about their sexual orientation, α = 0.84). The items were measured on a seven-point Likert scale, ranging from 1 = *not at all* to 7 = *every day* (Konishi et al., 2017). Mean scores of the variable were calculated and higher scores indicated higher levels of homophobic bullying perpetration. Cronbach’s alpha (α) was 0.84, suggesting that the items have relatively high reliability. Based on the exploratory factor analysis (EFA), a one-factor structure was revealed, and all items have factor loadings between 0.49 and 0.85.

#### Homophobic victimization

Homophobic victimization was measured using seven items asking students about their experiences with homophobic peer victimization at school (e.g., How often have you had someone saying negative things or teasing about your sexual orientation). The items were measured on a seven-point Likert scale, ranging from 1 = *not at all* to 7 = *every day* (Konishi et al., 2017). Responses to the items were averaged, with higher scores indicating greater homophobic bullying victimization. Cronbach’s alpha (α) reliability coefficient was 0.79, indicating that the items have good reliability. The EFA revealed a one-factor structure, and all items demonstrated acceptable loadings between 0.43 and 0.90.

### Data analytical plan

On a preliminary fashion, descriptive statistics (see Table 1) and correlational analyses (see Table 2) were performed to examine the intercorrelations between the variables included in this study.

**Table 2.** School characteristics and experiences by sexual orientation and sex.

	Adult acceptance		Peer acceptance		Homophobic victimization		Homophobic perpetration	
	*M*	*SD*	*M*	*SD*	*M*	*SD*	*M*	*SD*
Sexual orientation								
Completely heterosexual	5.52	1.20	4.97	1.34	1.27	.68	1.31	.68
Mostly heterosexual	5.28	1.75	4.07	1.58	1.41	.82	1.09	.18
Bisexual	5.09	1.05	3.97	1.40	1.63	.58	1.31	.68
Mostly homosexual	5.83	.76	4.33	.44	2.95	.58	1.05	.08
Completely homosexual	5.25	1.13	4.75	1.09	3.21	1.64	2.00	2.00
Not sure	4.93	1.83	4.26	1.84	1.88	1.67	1.16	.37
Sex								
Girls	5.34	1.28	4.62	1.52	1.40	.73	1.07	.22
Boys	5.52	1.19	4.89	1.29	1.34	.76	1.42	.81

Then, to test the study’s hypothesis, path analyses using Mplus (version 8; Muthén and Muthén, 2017) were conducted. The following observed variables were included in the saturated model: adult acceptance of sexual diversity, peer acceptance of sexual diversity, students’ homophobic bullying perpetration, students’ homophobic peer victimization, sex, and grade. The endogenous variables in this study were students’ homophobic perpetration and students’ homophobic peer victimization. Adult and peer acceptance of sexual diversity were the exogenous variables, whereas sex and grade were included as covariates. As such, the statistics, based on the regression analyses, were used to examine the relations between the exogenous variables (adult and peer acceptance of sexual diversity) and the endogenous variables (homophobic perpetration and victimization). The model was fully identified.

## Results

First, descriptive statistical results were obtained to examine students’ reports of homophobic bullying behaviors, students’ experiences with homophobia, and their perception of adult and peers’ acceptance of sexual diversity (see Table 2). Overall, the majority of the students reported a moderate to great level of adult acceptance of all sexual orientations (*M* = 5.64, *SD* = 1.45) as well as student acceptance of all sexual orientations (*M* = 4.68, *SD* = 1.77) in schools. Regarding students’ homophobic perpetration and peer victimization, overall, students reported a low level of engagement in homophobic behaviors (*M* = 1.29, *SD* = 0.67) and a low level of experiences with peer victimization (*M* = 1.37, *SD* = 0.79). As shown in Table 2, experiences of homophobic peer victimization were the highest among those who are completely homosexuals (*M* = 3.21, *SD* = 1.64) compared to those who are completely heterosexuals (*M* = 1.27, *SD* = 0.68). On average, the level of homophobic perpetrations was also the highest among those who are completely homosexuals (*M* = 2.00, *SD* = 2.00).

Next, correlational analyses were conducted to assess the intercorrelations between the variables included in the study (see Table 3). The findings revealed a significant negative relation between homophobic perpetration and adult acceptance of sexual diversity, *r* (219) = −0.172, *p* < .01, as well as between homophobic victimization and adult acceptance of sexual diversity *r* (219) = −0.344, *p* < .01. Although there was not a significant association between homophobic perpetration and peer acceptance of sexual diversity, homophobic victimization was significantly and negatively related to peer acceptance of sexual diversity, *r* (219) = −0.260, *p* < .01.

**Table 3.** Intercorrelations among study variables.

Variable	1	2	3	4	5	6
1. Homophobic perpetration	1.00					
2. Homophobic victimization	.282**	1.00				
3. Grade	−.083	−.119	1.00			
4. Sex	.244**	−.043	−.073	1.00		
5. Peer acceptance of sexual diversity	−.102	−.260**	.007	.106	1.00	
6. Adult acceptance of sexual diversity	−1.72**	−.344**	−.032	.099	489**	1.00

*Note*. **p < .01 (one-tailed).

*n* = 219.

A path analysis using Mplus 8.6 was conducted to examine the effects of adult acceptance of sexual diversity on students’ engagement in homophobic bullying behaviors and on students’ experiences with homophobia. As shown in Table 4, the findings revealed that adult acceptance of sexual diversity was significantly and negatively associated with both homophobic perpetration, *β* = −0.221, *t* (9) = −2.68, *p* = .002, and homophobic peer victimization *β* = −0.193, *t* (9) = −3.05, *p* = .007, controlling for grade and sex. However, peer acceptance of sexual diversity was not significantly associated with either homophobic perpetration *β* = −0.037, *t* (9) = −0.518, *p* = .605, or victimization, *β* = −0.123, *t* (9) = −1.710, *p* = .087. Figure 1 presents the path analysis model. Thus, the results suggest that students who perceived higher acceptance of sexual diversity among adult school members reported lower level of homophobic perpetration and lower level of homophobic victimization.

**Table 4.** Multivariate regression analysis for adult acceptance predicting homophobic perpetration and victimization.

Dependent variables	Independent variables	Estimate	SE	p-Value
Homophobic perpetration	Adult acceptance of sexual diversity	−.221	.071	.002
	Peer acceptance of sexual diversity	−.123	.072	.087
	Grade	−.115	.064	.073
	Sex	−.035	.065	.593
Homophobic victimization	Adult acceptance of sexual diversity	−.193	.071	.007
	Peer acceptance of sexual diversity	−.037	.072	.605
	Grade	−.074	.064	.242
	Sex	.266	.062	.000

**Figure 1.**
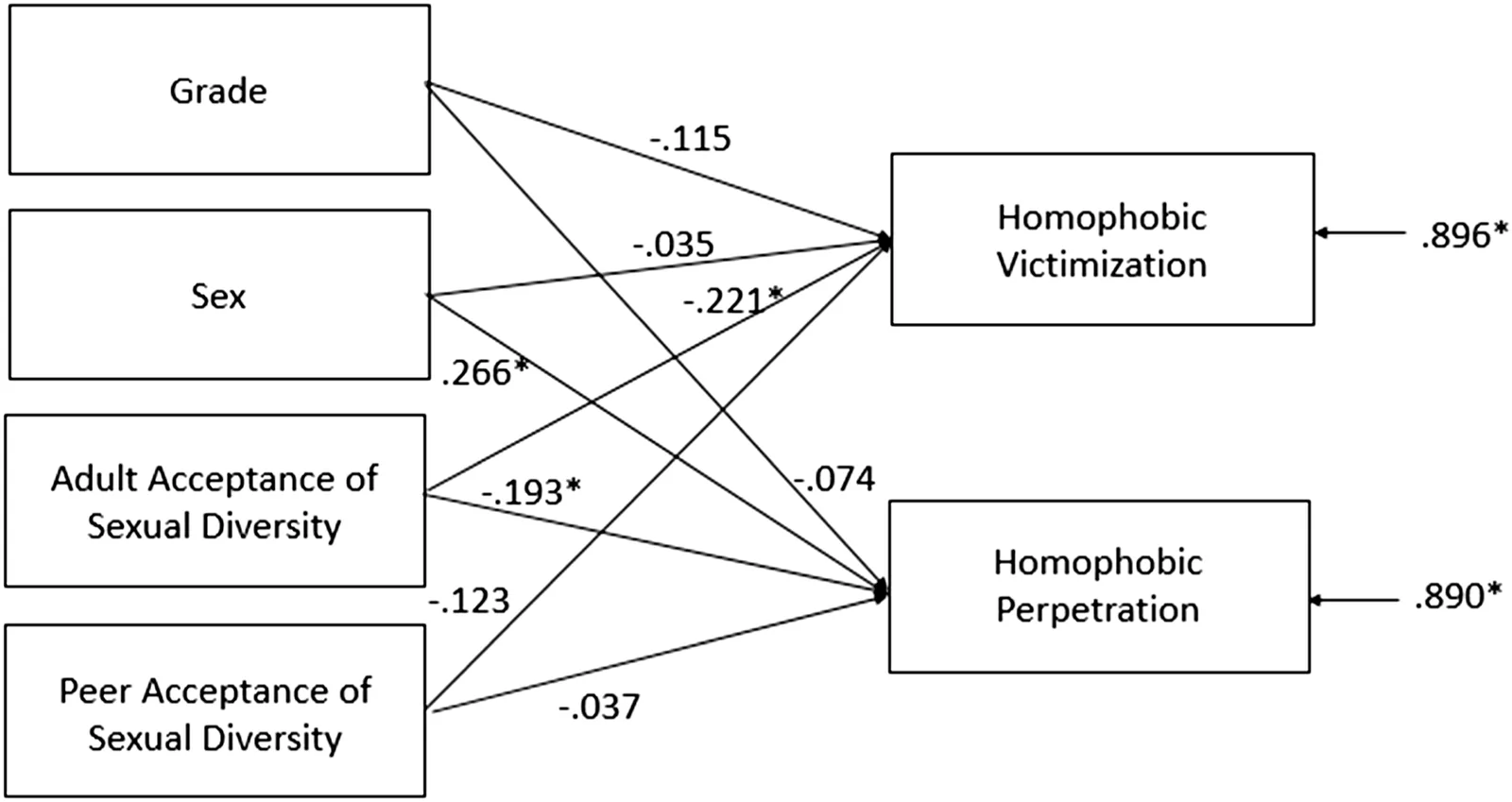
Path analysis model. *Note.* Estimates are standardized coefficients beta. *p < .05.

## Discussion

The current study examined the importance of peer and adults’ acceptance of sexual diversity in relation to students’ homophobic perpetration and homophobic peer victimization. Our preliminary findings demonstrated that while heterosexual youth reported experiences of peer victimization, sexual minorities (e.g., completely homosexuals) experienced a higher level of victimization than their heterosexual counterpart. Therefore, it can be understood that homophobic victimization is a cause of concern for students of various sexual orientations, but particularly for sexual minority youth. Given the negative consequences of homophobia on students, there is a substantial need to examine the importance of a supportive school climate for youth, especially for those who engage in homophobic behaviors and those that are victims.

Our results provided evidence that adult acceptance, more than that of peer acceptance, of sexual diversity may help in reducing students’ homophobic behaviors in schools. Our finding on adult acceptance and homophobia echoes previous research (Bhana, 2012; Olweus and Limber, 2010). According to Olweus and Limber (2010), it is important to create a school climate characterized by warmth and involvement from adults, and that the attitudes of teachers towards bullying and victimization have implications for the extent of bullying involvement in the school or class. As such, it must be understood that adults in schools play a pivotal role in working towards social equity and improving the experiences of victimized youth in schools (Bhana, 2012). Our finding is significant given that the literature indicates that perceived adult acceptance of sexual diversity is important for youth’s sense of belonging in schools and feelings of safety (e.g., Green et al., 2021). However, contrary to expectations, peer acceptance of sexual diversity was not significantly associated with homophobic victimization and perpetration in the present study. To explain our unexpected findings, past research may help provide insights. Previous studies have revealed that social support from peers and family alone may not be sufficient to protect victimized adolescents from negative mental health outcomes (Rothon et al., 2011). Similarly, peer support has been found to negatively predict self-perceived academic competence among adolescents involved in bullying (Ma et al., 2009). Their findings suggested that friends of perpetrators were more likely to have a negative influence and engage in problematic behaviors, while victims may struggle to receive adequate support from their limited social circle. As for victims who may have a few friends, the victims’ friends may also be at a risk of experiencing psychosocial difficulties (Eisenberg et al., 2005; Ma et al., 2009), making it difficult for victims of bullying to receive adequate support from their friends. In light of our findings on peer acceptance, it is important to consider the role of allyship in cultivating supportive school environments. Allyship involves purposeful effort by individuals to fight against injustice and promote fairness (Miller, 2024). Promoting peer allyship can empower students to actively stand against the homophobic behaviors and reframe their common misconception and stereotypes about sexual minority communities. While peer engagement is crucial for fostering a sense of community and belonging (Crooks et al., 2021; McBeath et al., 2018), adults in schools have a unique role in setting the tone of allyship and building supportive peer relationships. Teachers and school staff can use their positions of privilege to model inclusive behavior and openly challenge discrimination (Miller, 2024). Such modeling not only helps to create a more inclusive school culture but also encourages students to be a part of the discourse and efforts to support their peers, particularly those from marginalized groups.

However, given that adolescents are more likely to experience victimization from their peers and that perpetrators are influenced by their friends, support from adults becomes important to reduce homophobic behaviors among peers. As suggested by previous research, such support from adults such as teachers maybe needed to dissipate the negative influences from friends and enhance the positive peer relations among adolescents (Ma et al., 2009). Active support from school members is needed to protect students from bullying and its harmful effects as adolescents may be more likely to turn to adults in their school for acceptance and support (Rothon et al., 2011). The findings from the studies along with our finding imply that factors other than peer acceptance may play a more influential role in determining homophobic behaviors. This may include individual attitudes, societal norms, and other contextual factors. Furthermore, our findings were specific to homophobic behaviors and acceptance of *sexual* diversity, whereas past research on peer support studied bullying and support more broadly (D’Urso and Pace, 2019). More research is needed to investigate the role of peer acceptance and homophobic victimization and perpetration as our results may point to the nuances in the function of peer acceptance. Nevertheless, our study suggested that garnering adult support, specifically adult acceptance of sexual diversity, can play a more key role in countering homophobic behaviors in schools.

### Implications from the present study

There are several implications of our study concerning enhancing the positive experience of youth who experience homophobia in school through adult support. Our results suggest that adults’ acceptance of sexual diversity is a significant predictor of perceived discrimination among students. This highlights that in addition to having peers as a valuable source of support, adults in schools are also key figures who can shape the feelings of safety and acceptance felt by students of diverse sexual orientation. A key implication concerning school personnel—including teachers, administrators, practitioners, and other staff – is that they need to be aware of their impact on students, particularly sexual minority youth. These adults in schools should take on a systems-approach by understanding that interventions need to target the homophobic and heteronormative school environments that are home to the students, and not the sexual-minority youth themselves (Abreu et al., 2021). Leaders and executives both at the school level and the school board level should enforce the implementation of workshops and trainings that equip teachers with the awareness and tools to foster a school environment that is accepting of sexual diversity. These implementations and practices can be further mandated by policy makers even beyond the school board level. Further, considering the positive implications of an accepting school climate, safe school policies and programs such as GSA, the Olweus Bullying Prevention Program (OBPP), nondiscriminatory policies, and inclusive curricula should be implemented to promote inclusivity and support for students who experience homophobia (Black et al., 2012). For instance, the OBPP emphasizes a positive school environment, including warmth and involvement from adults, as a key element to reduce bullying (Olweus, 2010). The implementation of OBPP in schools has revealed marked reduction in bullying and victimization, anti-social behavior and higher school satisfaction, and positive social relationships among students in Norway (Olweus, 1995, 2010). Such evidence-based intervention programs must be considered to support anti-bullying initiatives in schools and classrooms and make reference to homophobia and sexual orientation. However, implementing such evidence-based interventions is not sufficient without the commitment and motivation of adults, in particular teachers, parents, and other school personnel (Olweus and Limber, 2010). Thus, the implementation of these programs is only meaningful and of quality if adults are trained to address issues related to homophobia and willing to pro-actively create a safe and positive learning environment for students. Special attention to adult acceptance of sexual diversity is warranted for efforts to reduce homophobic peer victimization in schools.

## Limitations and directions for future research

This study is not without limitations. Given the lack of psychometrically sound measures assessing adult and peer acceptance of sexual diversity, one limitation to address in this study is the use of single item to assess adult and peers’ acceptance of sexual diversity. Single-item measures are more vulnerable to ambiguous interpretations (Hoeppner et al., 2011). Future studies should utilize multiple-item measures to increase psychometric advantages. Additionally, the study did not consider the socio-economic status (i.e., of both students and school districts) and the presence of prevention programs in schools when examining homophobic victimization. It is also noteworthy to mention that the lack of ethnic/racial diversity in the sample limits the generalizability of the findings, as does the underrepresentation of sexual minority youth. Thus, findings may be specific to this community sample of predominantly white heterosexual adolescents in southern Quebec. Despite the sample being predominantly heterosexual youth, our study indicated that sexual minorities experienced higher levels of homophobic victimization, emphasizing the need to address this issue urgently. Future research should strive to include a larger sample with diverse racial backgrounds and a greater representation of sexual minorities, particularly homosexual and questioning youth who were underrepresented in this study. Although the complexity of one’s sexual orientation identity has been considered in this study, current inclusive thinking should also consider the gender identity of participants (e.g., cisgender, transgender, and non-binary). By incorporating a more diverse sample, we can gain a more comprehensive understanding of the experiences and challenges faced by different marginalized groups within the larger context of homophobic victimization. In this study, only homophobic behaviors and victimization among peers were assessed. However, future research should explore homophobia from both peers and adults such as teachers and school staff to inform more effective interventions and policies for mitigating related issues for victims and creating a supportive school environment.

Moreover, examining students who may belong to both the racial and sexual minority group is recommended to understand the intersectionality between race and sexual orientation. As such, researchers can explore the role of adult support for this specific group who may experience both racial and homophobic bullying. In addition to school support, future studies should consider family acceptance of sexual diversity in relation to adolescents’ experience with homophobic bullying, as well as the role of bystanders. While our study contributes to the understanding of individual-level associations, we recognize the need for future research to incorporate multi-level analyses to investigate the collective impact of individual-level and higher-level factors (e.g., diversity climate through inclusive policies and culturally responsive practices) on bullying prevention. This will provide a more holistic understanding of the complex dynamics involved in bullying.

## Conclusion

This study’s examination of acceptance of sexual diversity and students’ engagement in homophobic behaviors and their experiences with homophobic peer victimization has revealed some important findings regarding the influence of school members. The findings revealed that adult acceptance is significantly related to lower homophobic perpetration and victimization, highlighting the critical role of teachers, administrators, and school personnel in creating a safe environment. Given that schools mirror society, adults in schools have a crucial responsibility to promote acceptance of diversity. In view of the above, a greater recognition of this role is far-reaching for fostering an inclusive environment for students. As Olweus (1995) has emphasized, children have the right to feel safe and receive the same benefit from education, and homophobic bullying would undermine this right. Thus, we urge all school members to respect diversity and accept students with an open heart.

## Notes

1. Heterosexual societal standards refer to the norms and practices that prioritize heterosexuality as the dominant and default organizing principle, where any other forms of sexual expression or identity are considered deviant (Bhana, 2012).

2. It is important to note that while the term “gay” is accepted and used to describe someone who is attracted to the same sex, it can be used in a derogatory manner to insult and demean someone, implying that being gay is bad.
